# Quantile regression of microgeographic variation in population characteristics of an invasive vertebrate predator

**DOI:** 10.1371/journal.pone.0177671

**Published:** 2017-06-01

**Authors:** Shane R. Siers, Julie A. Savidge, Robert N. Reed

**Affiliations:** 1 Department of Fish, Wildlife and Conservation Biology, Colorado State University, Fort Collins, Colorado, United States of America; 2 U.S. Geological Survey, Fort Collins Science Center, Fort Collins, Colorado, United States of America; University of Arkansas Fayetteville, UNITED STATES

## Abstract

Localized ecological conditions have the potential to induce variation in population characteristics such as size distributions and body conditions. The ability to generalize the influence of ecological characteristics on such population traits may be particularly meaningful when those traits influence prospects for successful management interventions. To characterize variability in invasive Brown Treesnake population attributes within and among habitat types, we conducted systematic and seasonally-balanced surveys, collecting 100 snakes from each of 18 sites: three replicates within each of six major habitat types comprising 95% of Guam’s geographic expanse. Our study constitutes one of the most comprehensive and controlled samplings of any published snake study. Quantile regression on snake size and body condition indicated significant ecological heterogeneity, with a general trend of relative consistency of size classes and body conditions within and among scrub and *Leucaena* forest habitat types and more heterogeneity among ravine forest, savanna, and urban residential sites. Larger and more robust snakes were found within some savanna and urban habitat replicates, likely due to relative availability of larger prey. Compared to more homogeneous samples in the wet season, variability in size distributions and body conditions was greater during the dry season. Although there is evidence of habitat influencing Brown Treesnake populations at localized scales (e.g., the higher prevalence of larger snakes—particularly males—in savanna and urban sites), the level of variability among sites within habitat types indicates little ability to make meaningful predictions about these traits at unsampled locations. Seasonal variability within sites and habitats indicates that localized population characterization should include sampling in both wet and dry seasons. Extreme values at single replicates occasionally influenced overall habitat patterns, while pooling replicates masked variability among sites. A full understanding of population characteristics should include an assessment of variability both at the site and habitat level.

## Introduction

One of the key unanswered questions in ecology is how spatial structure of populations is influenced by ecological and demographic processes [1,2]. Variability in resource requirements and the distribution of those resources may result in the uneven distribution of demographic fractions across a heterogeneous landscape. Most studies on the spatial structure of populations occur across large-scale ecological gradients (e.g., latitude or altitude) and seek to identify broad patterns in behavior, morphology, life history, ecology or evolution. Sampling of a small number of populations at great distances and assuming monotonic changes between those populations may mask significant heterogeneity at finer spatial scales and make patterns appear more predictable at broader scales because effects of local heterogeneity are averaged out [3]. Averaging population characteristics over large areas can mask important localized population characteristics and dynamics such as variation in size distributions, reproduction, habitat affiliations, etc. Even seemingly homogeneous habitats may harbor structured subpopulations of vagile generalists [4,5], and population studies conducted at single sites may provide extremely biased conclusions [6].

Within populations, different demographic segments (e.g., sexes, age classes) may exhibit dissimilar ecological relationships (e.g., ontogenetic shifts in prey or habitat use), and the nature of those differences may have consequences for population studies including those involving climate change and population and habitat viability analyses. With respect to invasive species, smaller age classes containing greater numbers of individuals may more likely be accidentally transported, while mature individuals pose greater risk of reproduction upon relocation and continued recruitment during eradication efforts. Accounting for variation in demography among sites and habitat may have consequences for success of proposed management interventions.

**Fig 1:**
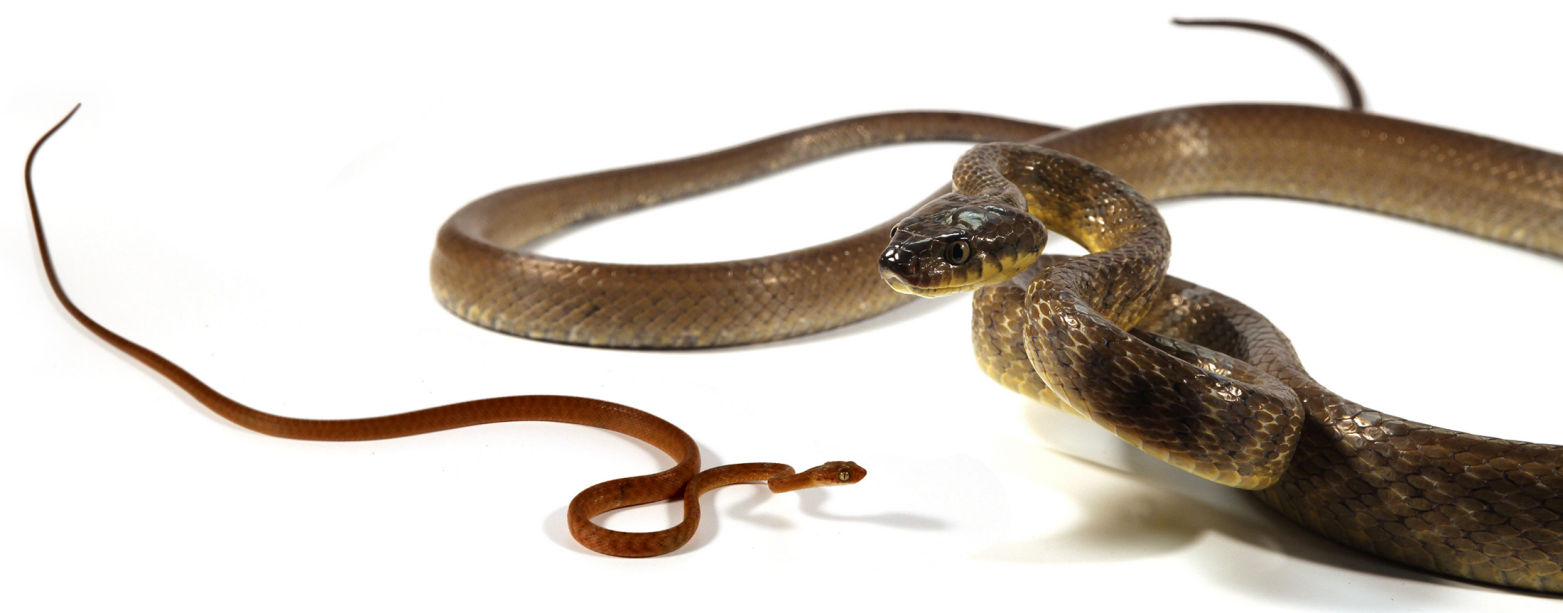
Hatchling and mature Brown Treesnakes. Photograph by S. R. Siers.

Sixty years after the introduction of Brown Treesnakes (*Boiga irregularis*), the island of Guam offers the opportunity to investigate the spatial variability of an invasive predator within and among a diversity of habitat types on a relatively small geographic scale. After hatching, a Brown Treesnake may undergo over a 600% increase in length and a 400-fold increase in mass (e.g., Fig 1). During growth, Brown Treesnakes undergo a pronounced ontogenetic shift from a diet consisting almost exclusively of ectothermic prey (small lizards) to endotherms (birds and mammals) as adults [7–9]. Because current control technologies rely on rodents as trap or toxicant lures, the strong preference of juvenile snakes for small lizard prey renders these tools largely ineffective against snakes in smaller size classes [10,11]. For this reason, along with a snake’s transition into maturity as it grows [12], snake body size is the individual characteristic of greatest importance with respect to its ecology and management. Intraspecific differences in snake body size may result in ontogenetic shifts in habitat use [13] as they target size-appropriate prey in a heterogeneous landscape. Along with body size, body condition can influence a snake’s reproductive ability [14] and susceptibility to control tools, e.g., [10–11], and therefore is also germane to variability within and among Guam’s diverse habitats.

Since the 1980s and 1990s, e.g., [7,12,15,16], little effort has been dedicated to understanding the landscape-level ecology of the invasive population of Brown Treesnakes on Guam. Samples from prior demographic studies of Brown Treesnakes have either been based on small numbers of sites [17], pooled by large ecoregions [8,18], or classified by broad habitat type (forest vs. non-forest) [16]. These samples were obtained from museum collections [8,18], road-cruising for live and road-killed snakes [17] or by combined visual searching, trapping, collection of road-kills, and public donation [16], all of which involve inconsistent sampling biases that make precise, direct comparisons among samples difficult. Although objectives for the management of this damaging invasive species include landscape-scale suppression, little is currently known about how variation in Brown Treesnake population characteristics affects prospects for management success across Guam’s heterogeneous landscape.

Here we report on one of the most comprehensive and standardized assessments of the geographic variability of any snake population, with the intent of characterizing the extent to which Brown Treesnake size distributions and body conditions vary throughout the landscape of Guam. Given the relative importance of snake body size and condition, and the potential for population-level variability in these traits among Guam’s diverse habitats, we endeavored to answer the following research questions: 1) Does Brown Treesnake body size vary by site, habitat and sex? 2) Does body condition vary by site, habitat and sex? 3) Are there seasonal effects on size distributions and body condition by site, habitat and sex?

## Methods

### Study sites

The island of Guam is situated in the Philippine Sea roughly between New Guinea and Japan (13.2 to 13.7° N and 144.6 to 145.0° E). Although relatively small (∼540 km^2^), Guam is topographically and ecologically diverse, ranging from a wet northern limestone plateau historically dominated by moist, broadleaved evergreen forest, to a drier southern region with rolling hills and mountains of volcanic origin, largely comprised of savanna vegetation. Guam’s climate is characterized by a warmer wet season running from July through November and a cooler dry season from December through June. While forest habitats remain green year-round, southern savannas and sites dominated by exotic *Leucaena leucocephala* (“Tangantangan”) may become relatively arid and fire-prone during the dry season.

**Fig 2:**
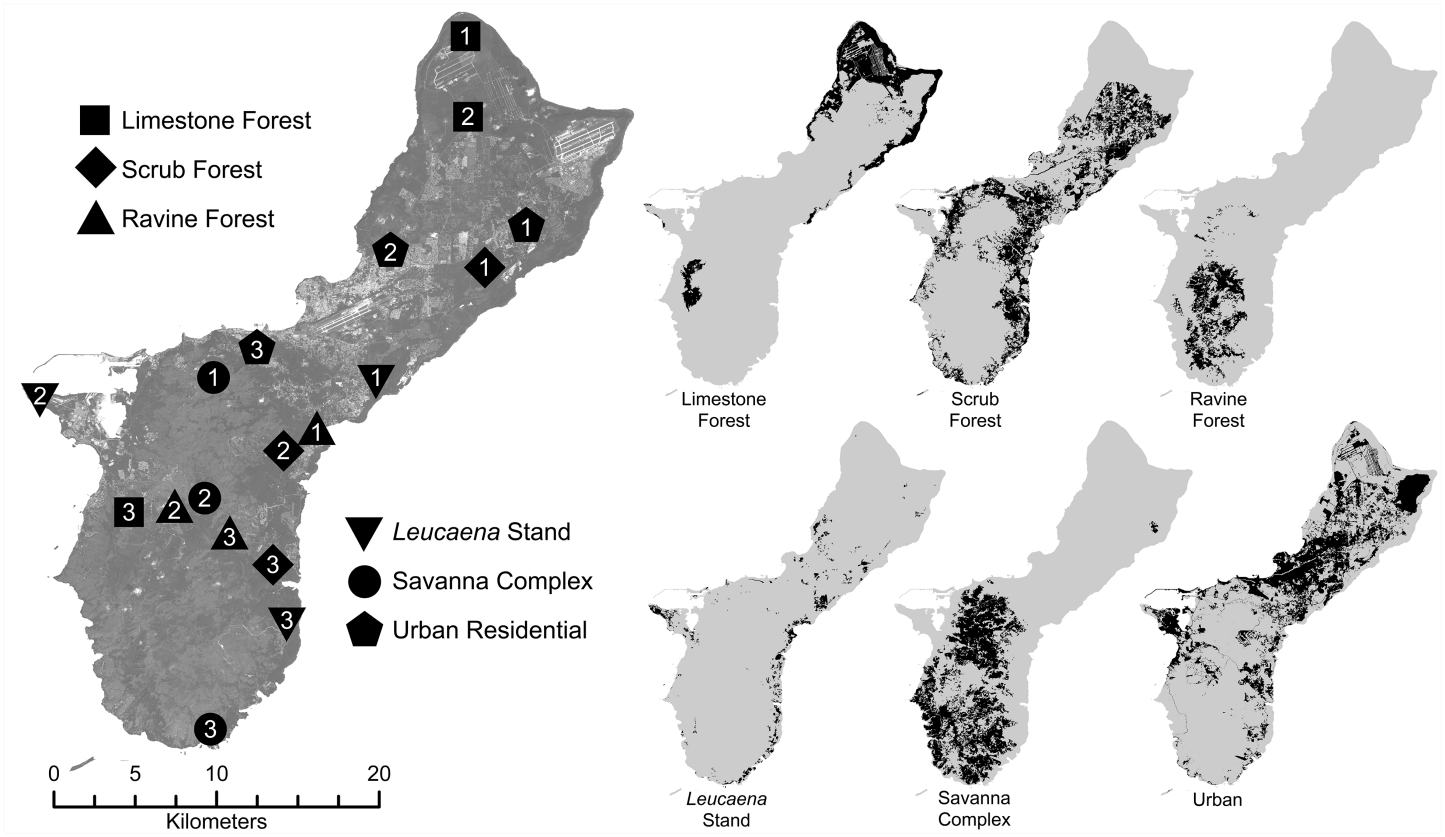
Habitat classification map of Guam. Depicts the distribution of the six target habitat types and the 18 sampling locations (after the classification of Liu and Fischer, 2005).

We selected three study sites within each of six habitat types as classified by Liu and Fischer [19] following the nomenclature of Mueller-Dombois and Fosberg [20]. Sites were dispersed across the majority of Guam’s geographic extent and ground-truthed to contain large uninterrupted tracts representative of the respective habitat type (Fig 2).

Permissions to conduct this field study at sites LIM1, LIM2, LIM3, SCR1, RAV2, LEU1, LEU2, SAV1, and SAV2 was granted by the Naval Facilities Engineering Command, Marianas. Permissions for sites on municipal lands were granted by the respective village mayors: URB1 (Yigo), URB2 (Dededo), URB3 (Agana), RAV1 and SCR2 (Yona), SCR3 (Talofofo), and LEU3 (Inarajan). Permissions for work at RAV3 and SAV3 were granted by the management of Onward Talofofo Golf Course and the University of Guam College of Natural and Applied Sciences, respectively.

### Habitat types

Limestone forest (code: LIM), 13% of Guam’s land cover, is characterized by moist, broadleaved evergreen forest of predominantly native species on elevated limestone plateaus. Scrub forest (SCR) is variable and composed of primarily non-native species resulting from a long history of human disturbance. It covers 23% of Guam’s land mass and comprises 58% of total forest cover. Ravine forests (RAV), 8% of Guam’s habitat, are low-lying areas surrounding flowing and ephemeral watercourses, and are primarily moist green forests containing higher proportions of palms, bamboos, and *Pandanus* spp. *Leucaena leucocephala* (LEU) is an introduced species providing excellent habitat for Brown Treesnakes. Nearly all forested areas on Guam have some amount of *Leucaena*, particularly at forest edges; however, in some areas it forms nearly monotypic stands that cover 3% of Guam’s land area. Savanna complex (SAV) is a mosaic dominated by grasses with emergent shrubby vegetation and erosion scars, and comprises a significant proportion of Guam’s southern region (21% of Guam’s total area). Urban built-up and landscaped areas, 27% of Guam’s habitats, include industrial, commercial and residential areas. For reasons of consistency, access, and public awareness, we elected to concentrate our surveys in and around urban residential (URB) areas. Together, these six habitat types comprise 95% of Guam’s land cover.

### Survey methods

We employed visual survey methods to sample snake populations at the eighteen selected sites. Visual surveys provide low yield per unit effort when compared to trapping, but samples are more representative of the population and exhibit less size bias [21]. Surveys commenced at sunset and were conducted for three to four hours, which includes much of the peak activity period of Brown Treesnakes [22,23]. These trained searchers, equipped with powerful headlamps, followed habitat edges at roughly 0.5 km per hour, examining all visible vegetation and non-vegetative structure for the presence of snakes. Forest habitats were surveyed from road or trail edges. Savanna searches included road edges, footpaths, and trackless searches throughout the habitat mosaic, including edges of erosion scars. Urban searches were conducted by searching residential yards, examining all structures and vegetation for the presence of snakes; yards were separated from large forest tracts by at least one paved road, as Brown Treesnakes tend to avoid crossing roads [24,25]. Searchers stopped searching when encountering habitat formations inconsistent with the search objectives and resumed searching upon returning to representative habitat. Survey effort (minutes) was recorded, along with counts of potential Brown Treesnake prey items observed while searching for snakes. Observation rates of key prey items for larger snakes (small mammals, birds and eggs, and *Anolis carolinensis* lizards) were calculated as number of sightings per 100 hours of search effort.

### Sampling objectives

To obtain enough data to accurately describe size distributions, we collected a target sample of 100 snakes from each site, for a total of approximately 1,800 snakes. To minimize the potential for bias resulting from short-term population dynamics or seasonal effects, sample sizes were balanced between wet and dry seasons, and each season was sampled in at least two quarterly bouts. The one exception was the second limestone forest replicate (LIM2), which was sampled in one relatively continuous effort due to impending construction of a snake-proof barrier; in this case, 90 snakes were collected in the wet season and 10 in the dry season.

### Snake processing

Upon visual detection, an attempt was made to hand-capture the observed snake. Hand capture was conducted by gentle manipulation with snake hooks, tongs, and or a gloved hand. Following capture, searchers recorded time and location of capture, microhabitat characteristics, and morphometric data. Morphometric data include snout-vent length (SVL), obtained by stretching the snake along a flexible tape ruler, and weight using Pesola spring scales (Pesola AG, Baar, Switzerland). Captured snakes were transported to the U.S. Geological Survey Brown Treesnake Lab the following morning, where SVL and weight were re-measured to validate field measurements. Snakes were euthanized by blunt force trauma resulting in immediate destruction of the brain, followed by decapitation, and then necropsied. Sex was determined by examining internal reproductive morphology. All animal use was conducted in accordance with Colorado State University Institutional Animal Care and Use Committee Protocol #09-1436A. No additional permits or protocol reviews were required for this study.

### Body condition

We assessed linear, quadratic, third-, and fourth-order polynomial models for fit to a regression of the log of snake body mass (subtracting the mass of any stomach contents) against the log of snake SVL for all sampled snakes, including both sexes, with model selection performed by comparing Akaike Information Criteria (AIC) values. We scaled residuals from the top model so that values are in units of standard deviations from the predicted value.

### Quantile regression

While most snake population studies report mean, median or modal SVL values and standard deviations to describe variation in body size, e.g., [26], these measures can be poor descriptors of potentially complex size distributions and may mask meaningful features of those distributions. Quantile regression allows estimation and inference on quantiles of a distribution without assumptions of normality and equality of variance required for standard regressions on mean values [27,28], and has previously been used to model geographic variation in growth and body condition for fish populations [29,30]. Because different quantiles of Brown Treesnake body size may vary in meaningful ways with respect to interdiction and control (i.e., smaller quantiles are refractory to most control methods and larger quantiles constitute greater threats of reproduction), we determined that quantile regression was an appropriate approach to estimating the effects of habitat type, sex, and seasonal variation throughout the range of snake body length quantiles. We followed the same procedures to assess inter-quantile differences in body condition.

We generated quantile regression fits for every 2.5th percentile from the 0.05th to 0.95^th^ quantiles of SVL and CI values for each sex at each site using the following model:

$${\text{Q}}_{\text{Y}}(\tau |\text{X})=\beta int(\tau )+\beta sex(\tau )+\beta site_{\text{j}}(\tau )+\beta sex*site_{\text{j}}(\tau )$$ (1)

where Q was the quantile estimate of Y (either SVL or CI) at the τth quantile (from the 0.05^th^ to 0.95^th^ quantiles) given the data (X), and β*int* was the regression intercept term. We coded an orthogonal sum contrast for *site* (j = 1, …, 17 sites, with the 18^th^ obtained by subtraction) and a 0,1 treatment indicator for female and male sexes, respectively. We plotted the site-specific quantile estimate offsets so that female quantile estimates were compared to the average of all females at all other sites, while male estimates were compared to other males. We obtained quantile estimates with the linear quantile regression function ‘rq()’ from ‘quantreg’ package version 5.05 [31] for R version 3.0.2 (<http://cran.r-project.org>). Standard errors (SE) were estimated using the “rank” method, and 95% confidence intervals were estimated for β(τ) at ± 1.96*SE. To investigate habitat and seasonal effects on quantile estimates for SVL and CI, we pooled replicates by habitat type and added a season covariate along with sex*season and sex*habitat interaction terms:

$${\text{Q}}_{\text{Y}}(\tau |\text{X})=\beta int(\tau )+\beta sex(\tau )+\beta hab_{\text{j}}(\tau )+\beta seas(\tau )+\beta sex*hab_{\text{j}}(\tau )+\beta sex*seas(\tau )+\beta hab_{\text{j}}*seas(\tau )$$ (2)

where *hab* is a categorical covariate with orthogonal contrasts coded for *j* = 1, …, 5 habitat types (6^th^ obtained by subtraction), and 0,1 treatment indicators for *sex* (female, male) and season (dry, wet). We plotted SVL and CI offset estimates throughout the quantile range for each habitat type by sex, season, and sex* season interaction. These offsets represent the deviation of the sample quantile fits from the average quantile fits of the respective sex for the whole study population.

## Results

**Fig 3:**
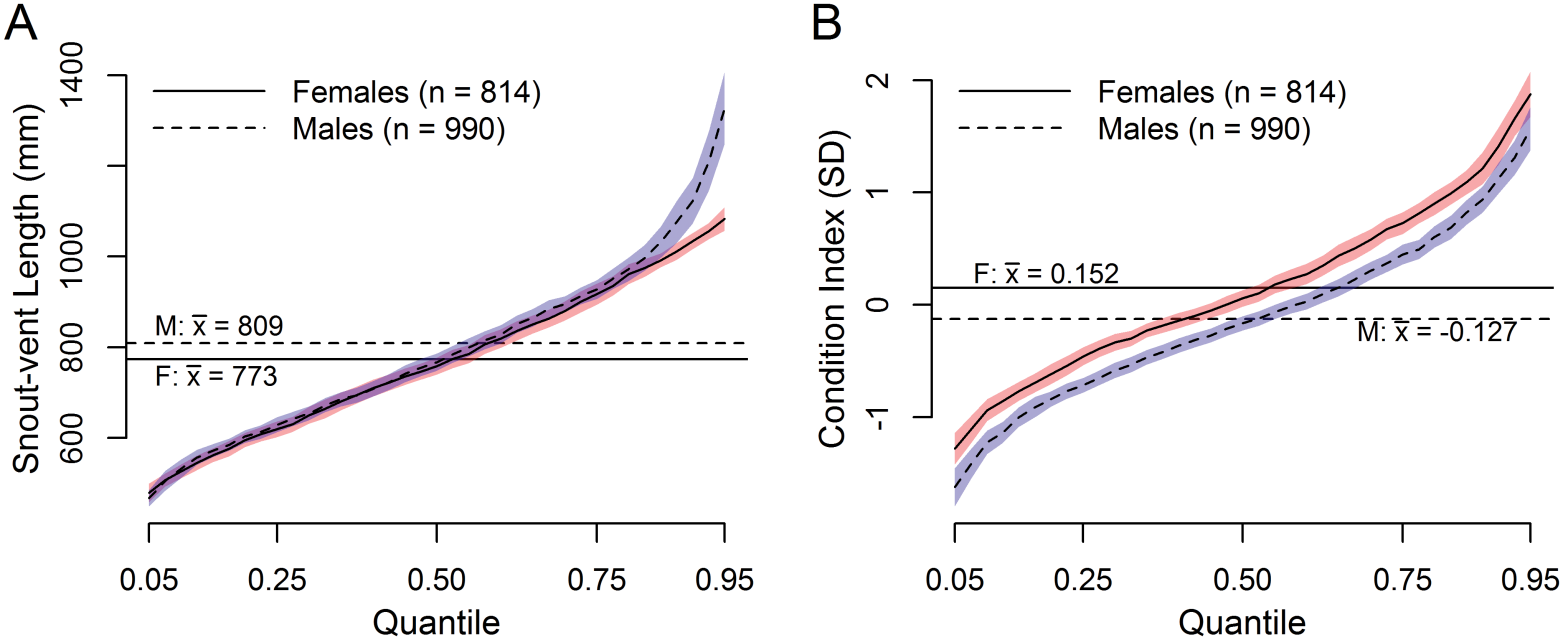
Quantile regression intercept values (cumulative distribution functions) comparing male and female body size (A) and condition index (B). Lines connect fit estimates for each quantile, and polygons represent the 95% confidence intervals for the estimates. Non-overlapping confidence polygons indicate significant differences. Horizontal lines indicate mean values of SVL and CI for each sex.

All sites were sampled over a period of one to two years, with surveys beginning on 22 March 2010 and continuing through 27 September 2012 (with the exception of LIM2 which was sampled on 18 May 2010 and from 1 September to 28 October 2010). At each of the 18 study sites, we captured a mean of 100 snakes (n = 99 to 104) for a total of 1,804 snakes (990 males and 814 females). Summary statistics on sample size, snake length, mass, and body condition are reported by sex in Supplementary materials (S1–S3 Tables), with these metrics further summarized by pooling them into forest (limestone, scrub, ravine, and *Leucaena*) and non-forest (savanna and urban) habitat types in S4 Table. The largest snake captured was a 1950-mm male weighing 2,478 g, and the smallest a 350-mm, 7-g hatchling male. Quantile regression of sex-specific size distributions for the entire sample revealed no significant differences in SVLs of sexes in all quantiles up until about the 0.85th quantile. At around 1000 mm SVL, quantiles of male snakes became drastically and significantly higher than those of females (e.g., 245 mm longer at the 0.95^th^ quantile; Fig 3A). The log(SVL) by log(mass) relationship was best approximated by a 4^th^-order polynomial function. The standardized residuals from this function (used as our CI values) showed adequate fit to a normal qqline, but with a slight right skew, with 95% of values being between -1.87 and 2.17. Quantile estimates of sex-specific body conditions (CI) showed a relatively normal cumulative distribution for both sexes, but with the female estimate an average of 0.26 standard deviations higher along the entire quantile range and non-overlapping confidence intervals (Fig 3B).

**Fig 4:**
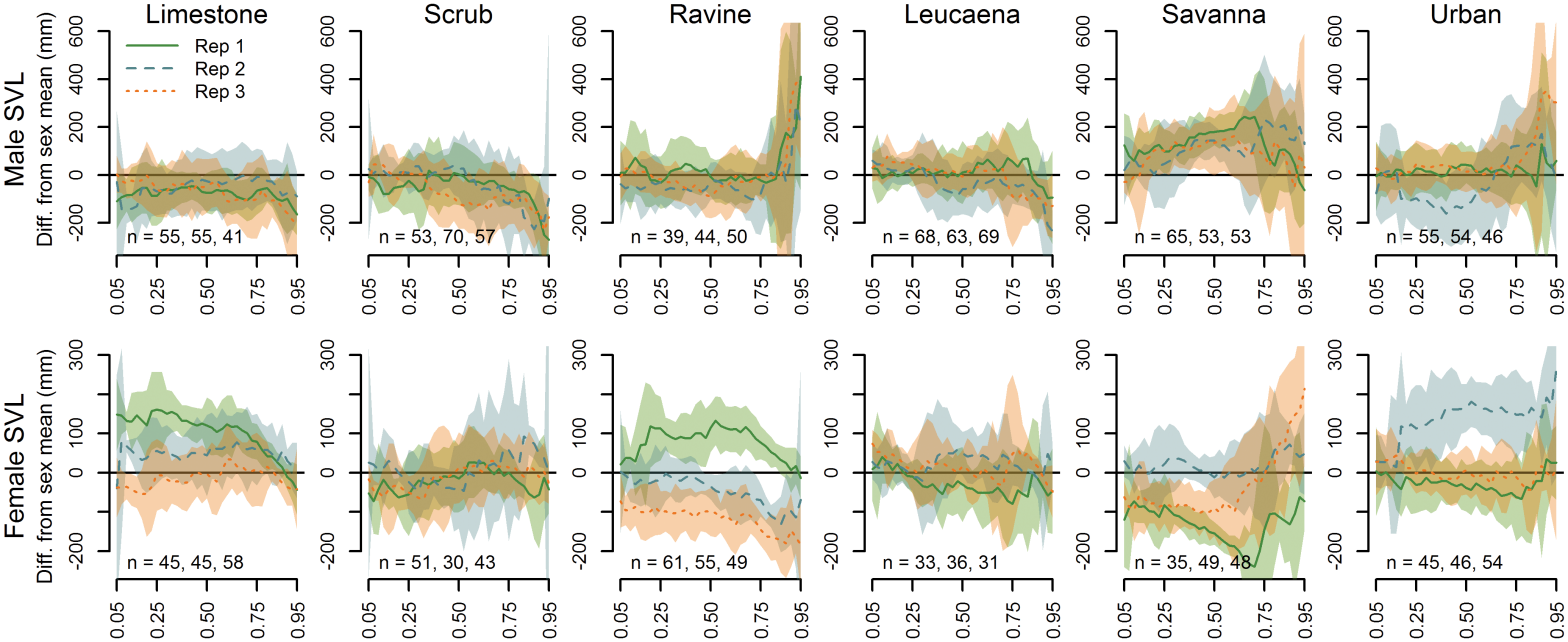
Comparison of male and female SVL quantile regression fits. Sex-specific offsets from the sex-specific quantile averages from all sites, for each of the 18 sampled sites (three replicate sites per habitat type) across the quantile range from 0.05 to 0.95 (x-axes). The horizontal line at y = 0 depicts the quantile average for the respective sex from all sampled sites. A segment of the confidence polygon that does not include the average line indicates a significant difference at the respective quantiles.

Interactions of sex and site (per Eq 1), grouped by habitat type, indicate the level of variability among replicates within and among habitat types in SVL (Fig 4). Size distributions for males were relatively consistent among sites within limestone, scrub, ravine, *Leucaena*, and savanna habitats. Females also showed relative consistency in scrub and *Leucaena* habitat but considerable variability, including extreme deviation of some replicates from quantile averages, was observed in the other habitats. More specific observations include, but are not limited to: particularly large males in the higher quantiles at all ravine sites and some savanna and urban sites (SAV2, URB3); overall low female size distributions at RAV3 and SAV1, a high mid-range of females at RAV1, and an overall large size distribution of females at URB2; and relatively large females in the smaller quantiles of LIM1 but average sizes in the larger quantiles. These results are also simplified in Table 1.

**Table 1:** Simplified summary of quantile deviations from size (SVL) means. Deviations from the quantile means, by sex, of all samples pooled (0 on the y-axis of Figs 4 and 6) for snakes in the lower quantiles (“Low”, ~0.05–0.25), median quantiles (“Mid”, ~0.25–0.75), and higher quantiles (“High”, ~0.75–0.95). The symbol “–” indicates SVL values lower than the mean, “=“ indicates similar to the mean, “+” indicates SVL values higher than the mean. Significance of deviations can be determined from confidence intervals in Figs 4 and 6.

Habitat	Sex	Replicate 1			Replicate 2			Replicate 3			Replicates Pooled		
		Low	Mid	High	Low	Mid	High	Low	Mid	High	Low	Mid	High
Limestone (LIM)	M	–	=	–	–	=	–	=	=	–	–	=	–
	F	+	+	=	+	+	=	–	=	=	=	+	–
Scrub (SCR)	M	=	=	–	=	=	–	=	–	–	=	=	–
	F	=	=	=	=	=	=	–	=	=	=	=	=
Ravine (RAV)	M	=	=	+	=	=	+	=	=	+	=	=	+
	F	=	+	=	=	=	–	–	–	–	=	=	–
Leucaena (LEU)	M	=	=	–	=	–	–	=	=	–	=	=	–
	F	=	=	–	=	=	=	+	=	=	+	=	–
Savanna (SAV)	M	+	+	=	=	+	+	=	+	=	=	+	+
	F	–	–	–	=	=	+	–	–	+	–	–	+
Urban (URB)	M	=	=	=	=	–	+	=	=	+	=	=	+
	F	=	–	=	=	+	+	=	=	=	=	=	+

**Fig 5:**
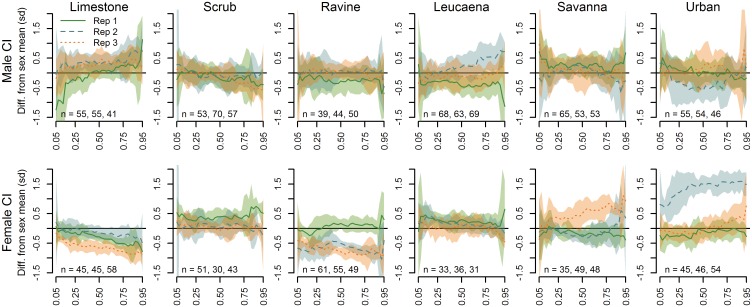
Comparison of male and female CI quantile regression fits. Sex-specific offsets from the sex-specific quantile averages from all sites, for each of the 18 sampled sites (three replicate sites per habitat type) across the quantile range from 0.05 to 0.95 (x-axes). The horizontal line at y = 0 depicts the quantile average for the respective sex from all sampled sites. A segment of the confidence polygon that does not include the average line indicates a significant difference at the respective quantiles.

Interactions of sex and site (Eq 1) with CI as the response variable (Fig 5) showed males with relatively consistent and “average” CI in scrub and ravine forest sites and female CI more consistent in limestone, scrub, and *Leucaena* habitat. In most cases, the behavior of the quantile fits was relatively parallel to the average values with the exception of LIM1 males and SAV3 and URB2 females. RAV2 and RAV3 females were in particularly low body condition, while URB2 females were in remarkably high body condition. These results are also simplified in Table 2.

**Table 2:** Simplified summary of quantile deviations from body condition index (CI) means. Deviations from the quantile means, by sex, of all samples pooled (0 on the y-axis of Figs 5 and 6) for snakes in the lower quantiles (“Low”, ~0.05–0.25), median quantiles (“Mid”, ~0.25–0.75), and higher quantiles (“High”, ~0.75–0.95). The symbol “–” indicates CI values lower than the mean, “=“ indicates similar to the mean, “+” indicates CI values higher than the mean. Significance of deviations can be determined from confidence intervals in Figs 5 and 6.

Habitat	Sex	Replicate 1			Replicate 2			Replicate 3			Replicates Pooled		
		Low	Mid	High	Low	Mid	High	Low	Mid	High	Low	Mid	High
Limestone (LIM)	M	–	=	+	=	+	+	=	+	+	–	+	+
	F	=	–	–	=	–	–	–	–	–	–	–	–
Scrub (SCR)	M	=	=	–	=	=	=	=	=	–	=	=	–
	F	+	+	+	=	=	=	=	=	=	+	+	=
Ravine (RAV)	M	=	–	–	=	=	=	=	=	=	=	=	–
	F	=	=	=	–	–	–	–	–	–	–	–	–
Leucaena (LEU)	M	–	–	–	=	+	+	=	=	=	=	–	+
	F	+	=	=	+	=	=	=	=	–	+	+	–
Savanna (SAV)	M	+	=	+	=	=	–	+	=	=	+	=	=
	F	=	–	–	=	–	+	=	+	+	=	=	+
Urban (URB)	M	=	=	=	=	–	=	=	=	+	=	=	=
	F	–	=	=	+	+	+	=	=	+	=	+	+

**Fig 6:**
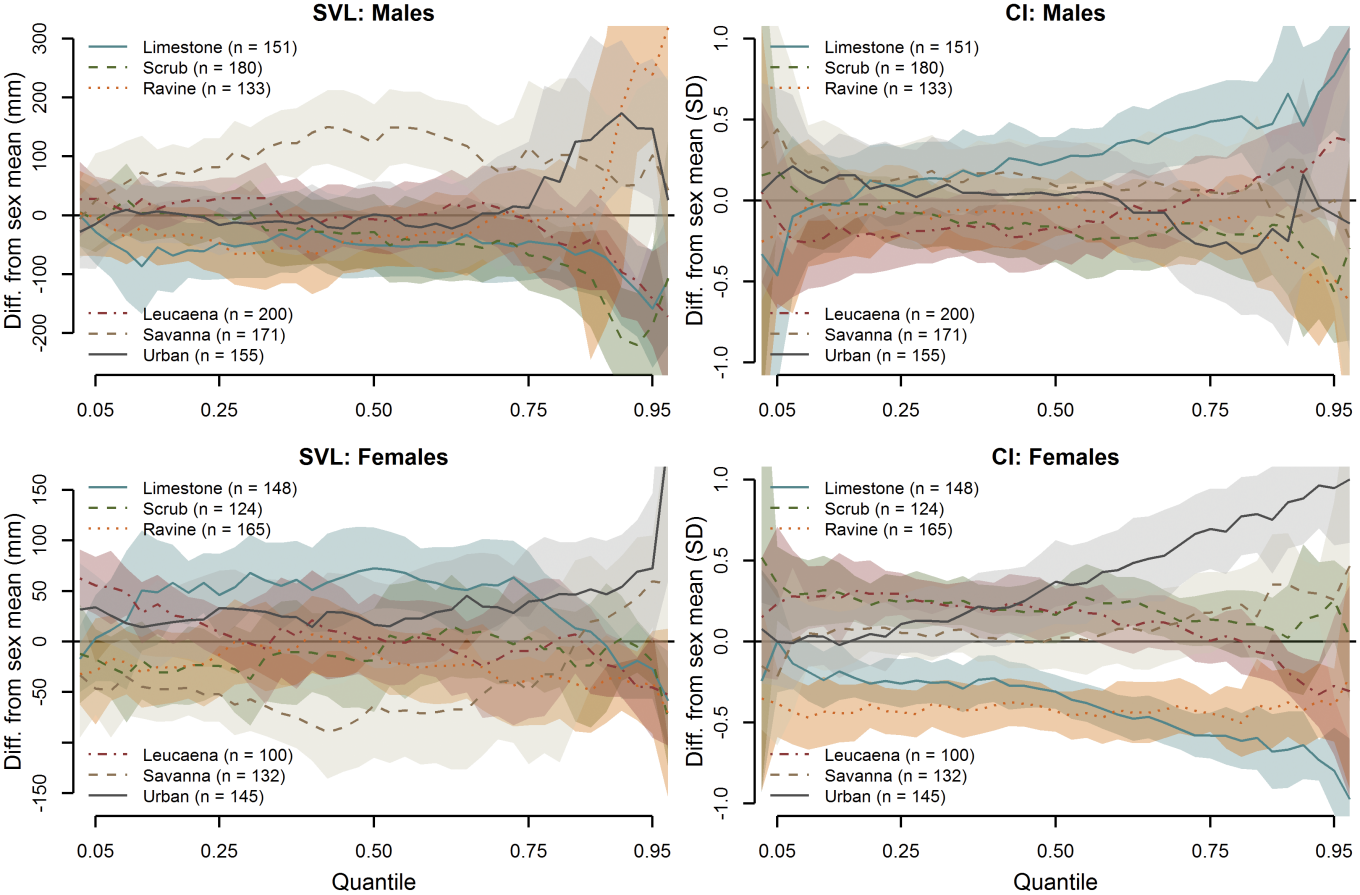
Quantile fits (offsets) by habitat type for male and female Brown Treesnake SVL and CI, indicating the variability among habitat types. The horizontal line at y = 0 depicts the quantile average for the respective sex from all sampled sites. A segment of the confidence polygon that does not include the average line indicates a significant difference at the respective quantiles.

The results of the SVL and CI quantile regression models that pooled captures by habitat type (Eq 2) describe the size distributions by habitat type and the variation in quantile estimates among habitats (Fig 6). Males in limestone forest tended to be below average length along the quantile range, particularly at the larger quantiles, while savanna males tended to be significantly larger than average across the quantile range. Males had noticeably higher than average lengths in the highest quantiles among ravine forest, savanna and urban sites, and below average lengths in those same quantiles for limestone, scrub and *Leucaena* forest. The higher quantile estimates for female SVL showed the largest snakes in savanna and urban habitat, similar to (but not as distinctive as) the pattern in male snakes, and ravine forest females tended to be well below average in the higher quantiles. Limestone forest females tended to be larger across the mid-quantile range, but smaller than average in the higher quantiles.

CI results under the same model (Eq 2, Fig 6) indicated a striking divergence in the body conditions of male and female snakes in limestone forest, with very high-CI males and low-CI females, particularly in the higher quantiles. While ravine forest males tended to be relatively average in body condition, with the exception of the highest quantiles, ravine forest females were in consistently poor body condition across the entire quantile range. Although urban male snakes were of relatively average body condition, the highest body conditions were observed in the upper quantiles of urban female snakes, superficially similar but opposite to the pattern of body condition in limestone forest snakes.

Some of these deviations in the pooled habitat samples appear to be driven by more extreme deviations within single replicates—e.g., the high SVL values in the upper quantiles of urban females result from the overall high female SVL values at URB2, while URB1 and URB3 are closer to the average.

**Fig 7:**
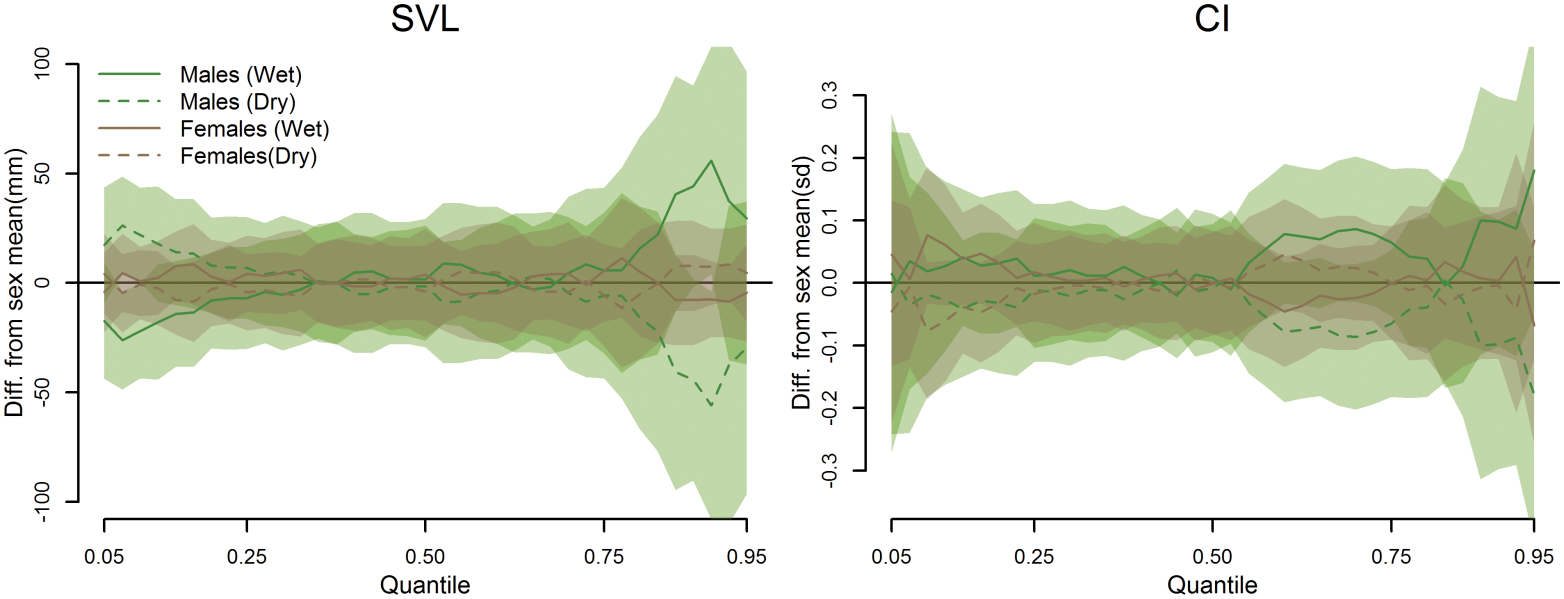
Seasonal differences in SVL and CI quantile estimates (offsets), averaged across all habitat types. The horizontal line at y = 0 depicts the quantile average for the respective sex from all sampled sites. A segment of the confidence polygon that does not include the average line indicates a significant difference at the respective quantiles.

The habitat model (Eq 2) also included a seasonal term with sex-by-season and habitat-by-season interactions. For both SVL and CI, there was little effect of season as a whole for either sex, with the exception of marginally larger and more robust (higher-CI) male snakes found in the higher quantiles during the wet season than in the dry season (Fig 7).

**Fig 8:**
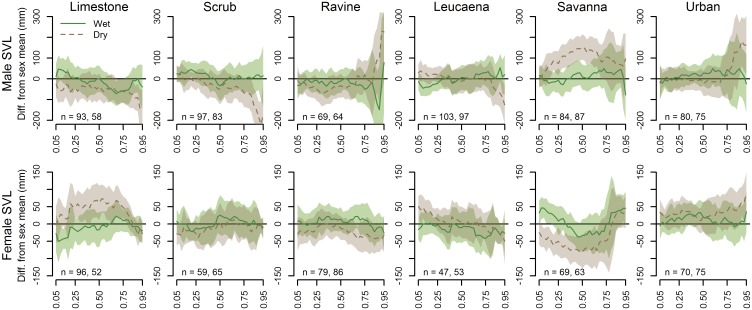
Wet season and dry season SVL quantile estimate comparisons, or sex-specific offsets from the sex-specific quantile averages from all habitats, for both sexes by habitat type. The horizontal line at y = 0 depicts the quantile average for the respective sex from all sampled sites. A segment of the confidence polygon that does not include the average line indicates a significant difference at the respective quantiles.

There was very little influence of season on SVL or CI of female snakes in any portion of the quantile range. However, more seasonal influence on SVL and CI was evident when examining the season-by-habitat interactions. In general, SVL distributions tended to more closely reflect the quantile averages during the wet season, with the most dramatic deviations from average occurring in the dry season (Fig 8). Scrub forest males skewed particularly low in the higher quantiles during the dry season, while savanna males were consistently large and ravine forest males spiked in size at the highest quantiles. The upper quantiles of urban males appeared larger than average only during the dry season. Limestone forest females tended to be relatively large across the mid-quantile range, and savanna females quite small, during the dry season.

**Fig 9:**
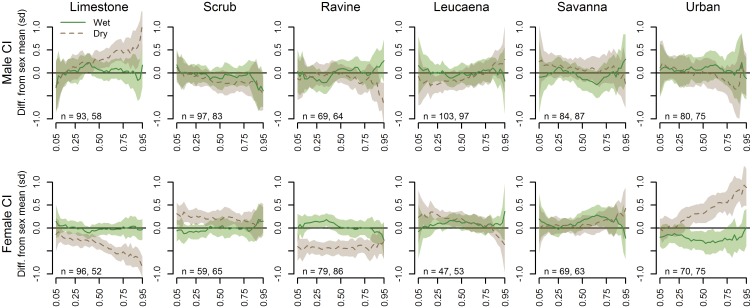
Wet season and dry season CI quantile estimate comparisons for both sexes, or sex-specific offsets from the sex-specific quantile averages from all habitats, by habitat type. The horizontal line at y = 0 depicts the quantile average for the respective sex from all sampled sites. A segment of the confidence polygon that does not include the average line indicates a significant difference at the respective quantiles.

A similar pattern of average distributions during the wet season and more extreme deviations during the dry season was evident in the CI habitat-by-season interaction estimates (Fig 9). In particular, a dry season spike in the higher CI quantiles was evident for limestone forest males and urban females, with below-average CIs for limestone and ravine forest females during the dry season.

Searchers logged a total of 2,338 survey hours. Observations of prey items for larger snakes were pooled into broad categories: small mammals (*Rattus* spp., *Mus musculus*, and *Suncus murinus*), birds (*Coturnix chinensis*, *Francolinus francolinus*, *Gallus gallus*, *Passer montanus*, *Streptopelia bitorquata*, and bird eggs), and Green Anole lizards (*Anolis carolinensis*). Small mammal sightings were far more frequent in savanna habitats, and birds and anoles more frequent in urban habitats, compared to relatively low sightings in forested habitats. Prey sighting rates are detailed in Table 3.

**Table 3:** Number of large snake prey items observed per 100 survey hours.

Habitat	Small Mammals	Birds and Eggs	Anole Lizards
Forest	0.06	3.00	0.32
Urban	5.66	112.01	30.83
Savanna	10.77	2.92	0.66

All data used in the analyses are provided as supporting information in S1–S3 Datasets, with metadata provided in S4 Dataset and R code provided in S1 Code.

## Discussion

### Does Brown Treesnake body size vary by site, habitat, and sex??

#### Lower size quantiles

We observed the greatest proportion of very small male snakes in limestone forest habitat and the fewest in savanna habitat. Very small females, however, were more prevalent in savanna habitat while the lowest quantiles of females tended to be larger in *Leucaena* habitat. With respect to the site-specific offset estimates, lower-quantile estimates were relatively average, particularly for small males, in the other habitats. Smaller snakes in the lower quantiles tended to be observed in outlier replicates, e.g., females from RAV3, SAV1, and SAV3 (Fig 4). Although sampling occurred over the course of one to two years in a seasonally-balanced fashion, it is possible that an excess of very small snakes at a few sites may be the result of conditions that led to high localized breeding and hatching rates.

#### Median size quantiles

Throughout the median quantiles, snakes in most habitats were relatively invariant in size. The most extreme deviations in median size occurred at only one or two sites within habitat classes (e.g., LIM1, RAV1 and 3, SAV1 and 3, and URB2 females). Pooling by habitat, median sizes for female snakes in limestone forest habitats were significantly higher than average while males were about average or slightly smaller than average. Conversely, median male snake size was significantly higher than average in savanna habitats, while females were significantly smaller (Fig 6). While we can offer no intuitive process that might drive such differences in the limestone forest habitat, it is possible that the skew toward larger males and smaller females at savanna sites may in part be due to the greater role of rodents in the diets of Brown Treesnakes in this habitat [7,32,33].

#### Higher size quantiles

The most striking divergence in snake sizes by habitat is that the highest quantiles of snakes are larger in savanna and urban habitats for both males and females, though there is much variability among replicates. This is likely the result of availability of larger prey such as rodents in savanna grasslands and human-commensal prey species (rodents, invasive birds, poultry) in urban residential areas ([7,33], Table 3). There is also a spike in male SVL at the highest quantiles in ravine forest. Ravine forests harbor abundant large frogs that may be preyed upon by larger snakes; however, observations of large frogs in stomach contents of snakes is relatively rare. While we captured more snakes of reproductive size in urban habitats, particularly URB2 for females, the lack of excess hatchlings in urban habitats (as would be indicated by lower estimates in the lower quantiles at urban sites) suggests that either young are not surviving, are more difficult to detect in this habitat, or oviposition is occurring in other habitats and residential areas may merely be transient feeding grounds. However, the presence of large females at SAV3 does coincide with the presence of smaller females in the lower quantiles.

#### Size differences by sex

Geographic variation in sexual size dimorphism resulting from variation in prey availability has been observed in Australian pythons [34], and it is possible that this phenomenon can be observed among habitat types varying in available prey types; juvenile male Brown Treesnakes grow at a faster rate than females (Bjorn M. Lardner, unpublished data), and it is plausible that faster-growing male snakes may be more successful at bridging the gap in prey size between small lizards and large grassland rodents, leading to increased growth of males and suppressed growth of females. However, this might lead one to expect particularly low body conditions for savanna females, and this does not seem to be the case (Fig 6). Emigration of nutritionally stressed individuals from savanna habitat may account for the deficit of mid-sized females without an obvious body condition pattern, but this would likely be evidenced by a greater male bias in sex ratios in savannas compared to other habitats; no such pattern was apparent in our data.

### Does body condition vary by site, habitat and sex?

Throughout the entire quantile range of CI values, females consistently exhibited higher body condition than males (Fig 3B). As metabolic demands of reproduction fall more heavily on female Brown Treesnakes, they may invest growth capital in energy stores [35,36] and a more robust somatic mass rather than body length; Savidge [16] observed a similar pattern among urban Brown Treesnakes on Guam and suggested this result supported the hypothesis that females are channeling growth resources into eggs or fat storage rather than length. There was generally more variability in body condition within savanna and urban habitats. Additionally, male snakes showed considerable variability among replicates in limestone and *Leucaena* forest while replicates for female snakes were variable in ravine forest. Female snakes demonstrated some of the most extreme highs (SAV3, URB2) and lows (LIM3, RAV2, RAV3) of body condition (Fig 5). Differences in body condition between males and females within a single site were sometimes extreme: LIM1 males showed a very broad distribution of CI values (high values in the upper quantiles and low values in the low quantiles) while females showed a more narrow distribution (higher values in lower quantiles and lower values in higher quantiles) averaged below the quantile intercept; at URB2, CI values for females were remarkably high across all quantile ranges while male CI values were mostly low, with this distinction being even more pronounced when considering the female baseline was higher than the male baseline. Among pooled limestone forest sites, the general trend was for higher CI variance and mean among males and a lower variance and below-average CI among females. This pattern was nearly reversed for urban habitats, with female CIs much higher than male CIs in the higher quantiles (largely driven by extreme values at URB2). While male CIs in ravine forests were average (RAV2 and 3) to low-average (RAV1), female CIs in RAV2 and 3 were consistently low, indicating ravine forests, along with limestone forests, provide relatively poor opportunity for amassing metabolic stores for reproduction by females (Fig 6), consistent with low prey availability.

### Are there seasonal effects on size distributions and body condition by site, habitat and sex?

Season appeared to have little influence on the sizes of snakes detected during night searches, with the exception of a marginal increase in the size of male snakes in the higher quantiles during the wet season (Fig 7). As with body condition, the majority of variability in seasonal SVL distributions occurred in the dry season, while wet season samples were relatively average (Fig 8). Most notably, there appeared to be a dry season depression in the higher quantiles of male snakes in the limestone, scrub and *Leucaena* forest types and an increase in the ravine forest, savanna and urban habitats, potentially indicating seasonal changes in habitat use or foraging activity of large males among these habitat types. The difference between mid-quantile SVLs for male and female savanna snakes, previously noted, seems to be primarily driven by differences that occurred in the dry season, though an explanation for this pattern is not evident. Occasional site-specific abundances of small snakes as evidenced by low estimates in the lower quantiles (e.g., RAV3, SAV1, SAV3; Fig 4) may be evidence of recent breeding activity. However, these do not appear to be associated with season, at least when averaged by habitat type (Fig 8). This is not surprising given that Brown Treesnakes on Guam do not exhibit notable seasonality in reproduction [12,37].

Body conditions of snakes can be sensitive to recent fluctuations in prey availability, which in turn may be driven by seasonal weather patterns [38,39]. We anticipated that the surge of primary productivity associated with increased precipitation during the wet season would lead to an increase in insects, in turn feeding the small lizards that have become the primary prey of Brown Treesnakes on Guam [7]; this would result in overall better body conditions for snakes in the wet season. Overall, season did not appear to have a consistent effect on snake body condition in general, with only a marginal increase in the highest quantiles of CI for male snakes (Fig 7). More distinct variation in CI was evident when observing seasonal changes by habitat (Fig 9). In general, wet season offset estimates for both sexes and all habitats tended to be close to zero, while the greatest deviations occurred during the dry season. The previously-mentioned patterns in limestone forest and urban CI appear to be primarily driven by CI extremes occurring during the dry season.

### General discussion

Other published accounts of geographic or ecological variation in population characteristics of snakes have demonstrated variation in: sexual size dimorphism (*Morelia spilota* [34,40], *Elaphe quatuorlineata* [41]); body size, age structure, and proportions of reproductive animals (*Pseudechis porphyriacus* [42], *Opheodrys aestivus* [43], *Liasis fuscus* [5], *Elaphe obsoleta* [44], *Natrix natrix* [45], *Crotalus oreganus* [46]); reproductive characteristics of females (*Thamnophis sirtalis* [47], *Hierophis viridiflavus* [48], *Vipera aspis* [49]); individual growth rates (*Thamnophis elegans* [50]); and microhabitat use (*Corallus grenadensis* [51]). However, many of these studies occurred over very large geographic expanses, e.g., [44,47], over distinct elevational gradients [50], or at a small number of sites [43,51]. Within the snake literature, our study is one of the first to use a controlled and balanced sampling design methodology to explicitly assess the amount of variability present among multiple replicates, habitat types, and seasons over a relatively small geographic expanse.

Spatial patterns of snake populations reflect behaviors and interactions of individuals and spatial arrangements of prey and other resources [52]. However, generalities concerning the process of habitat selection remain largely based upon conjecture [53], and much remains to be learned about the influence of local ecological conditions on the spatial structure of populations. Our results explore the level of variability within and among habitat types throughout the entire extralimital range of this costly invasive predator. Although we found ecological influences on size distributions and body conditions, microgeographic variation—as observed among replicates—often exceeded the level of variability observed among habitat types. Indeed, some deviations at single replicates were extreme enough to appear to drive habitat “patterns,” even when other replicates were relatively average in size or body condition distributions. Conversely, averaging over replicates exhibiting much variability may suggest that the habitat is relatively average, masking what may be important variation (e.g., female SVL at ravine replicates). Such decisions to pool or parse data from multiple geographic locations may result in differing conclusions. For these reasons we maintain that a thorough understanding of the population ecology of this and similar organisms should include sampling at multiple sites within habitat types and an explicit consideration of the variability within and among habitat types, particularly for factors with strong demographic or management consequences.

The single individual characteristic most important to modulating the behavior, ecology, and management challenges associated with invasive Brown Treesnakes is body size. This characteristic drives prey utilization [7], susceptibility to control tools [54], reproductive maturity [12], and may be associated with patterns of habitat use and prevalence of various size classes in habitat types [16]. As “capital breeders” [55], Brown Treesnakes must amass sufficient metabolic energy stores prior to successful reproduction [14]. Our results indicate significant variability in body size and body condition by site and habitat, which may be useful to managers planning and prioritizing intervention activities.

The presence of larger snakes in urban and savanna habitats is almost certainly related to the relative availability of larger prey items in those habitats, as evidenced by our prey sightings (Table 3) and the results of stomach contents analyses [7,33]. This pattern can be explained by two processes: 1) snakes growing into size classes requiring larger bird and mammal prey may range more broadly in search of appropriate prey and encounter more abundant resources in other habitats (resource selection); or, 2) snakes resident in habitat with larger prey may grow more quickly and to larger sizes, while snakes in prey-depauperate habitat may grow more slowly and exhibit reduced survival (differential growth and survival). In the absence of reliable snake aging techniques, our cross-sectional study design cannot distinguish between these two processes. In reality, they are not likely to be mutually exclusive and both may occur at some level, with the context of spatial distribution of local resource characteristics playing an important role.

We attempted to minimize short term effects such as weather events and prey or breeding pulses by sampling in multiple bouts over one to two years. However, it should be acknowledged that we cannot be certain whether our observed differences in population characteristics are stable or transient.

Despite the general trends observable in the results, this study indicates a high level of heterogeneity in population characteristics of invasive Brown Treesnakes throughout Guam; averaging population characteristics over larger areas would mask much of that heterogeneity. It appears that, with respect to prospects for snake suppression, there is little ability to make predictions about population characteristics in unsampled areas. Rather, knowledge of the possible range of population characteristics should be taken into account when making predictions about the outcomes of management interventions. Simulated suppression scenarios should incorporate the full range of variability reported here and explicitly consider sensitivity of predicted outcomes to plausible extremes of starting conditions.

There is no single “proper” scale for ecological studies [56] and those that include inference from multiple scales will inherently be more robust. For species with demographic strata that vary in ecological requirements such as habitat or prey types, it may be particularly important to assess and account for how those strata are distributed within a heterogeneous environment. Failure to consider microgeographic variability in population characteristics of such species may result in imprecise or erroneous inference or sub-optimal management outcomes.

## Supporting information

S1 Table Summary of snout-vent length (mm) by habitat type, replicate, and sex. (PDF)

S2 Table Summary of snake mass (g) by habitat type, replicate, and sex. (PDF)

S3 Table Summary of snake body condition index (CI) by habitat type, replicate, and sex. (PDF)

S4 Table Summary of snake body length, mass, and condition index pooled by forest and non-forest habitat types and by sex. (PDF)

S1 Dataset Original snake capture and morphometrics data. (CSV)

S2 Dataset Reorganized data for quantile regression models. (CSV)

S3 Dataset Prey observation data. (CSV)

S4 Dataset Metadata (column name keys) for S5–S7 Data. (DOCX)

S1 Code R code for quantile regressions and plotting. (R)
