# Supplementary material for: Quantile regression of microgeographic variation in population characteristics of an invasive vertebrate predator
Source: PLoS One. 2017 Jun 1;12(6):e0177671. doi: 10.1371/journal.pone.0177671 (PMC5453442; doi:10.1371/journal.pone.0177671)
Supplement: S1 Table — (PDF) [file pone.0177671.s001.pdf]

S1 Table. Summary of snout-vent length (mm) by habitat type, replicate, and sex.

		Replicate 1						Replicate 2						Replicate 3						Habitat Pooled					
Hab	Sex	N	Min	25%	Med	75%	Max	N	Min	25%	Med	75%	Max	N	Min	25%	Med	75%	Max	N	Min	25%	Med	75%	Max
LIM	M	55	502	712	818	945	1305	55	409	623	790	989	1597	42	391	567	723	851	1186	152	391	629	789	942	1597
	F	45	550	796	878	985	1072	45	378	691	801	965	1235	59	427	634	754	910	1128	149	378	676	836	965	1245
SCR	M	53	400	555	760	884	1123	70	506	621	762	879	1950	57	417	592	666	856	1209	180	400	601	716	871	1950
	F	51	400	583	742	899	1063	30	408	613	714	961	1404	43	460	590	770	913	1064	124	400	604	743	916	1404
RAV	M	39	356	723	878	996	1920	45	418	579	698	786	1549	50	354	517	582	788	1947	134	354	564	718	891	1947
	F	61	424	748	872	983	1083	56	398	629	722	835	1221	49	382	533	657	762	1167	166	382	611	750	877	1221
LEU	M	68	454	635	754	928	1395	63	466	656	761	925	1210	69	565	689	782	902	1170	200	454	664	765	922	1395
	F	33	501	625	727	827	1057	36	517	636	808	932	1140	31	567	648	774	930	1080	100	501	632	767	900	1140
SAV	M	65	375	650	796	922	1295	53	388	703	903	1174	1563	53	350	654	816	1015	1876	171	350	660	827	998	1876
	F	35	370	554	602	724	1030	49	505	657	745	906	1143	48	393	554	661	885	1346	132	370	571	694	866	1346
URB	M	55	408	636	775	912	1565	54	402	652	804	1207	1687	46	501	683	794	954	1779	155	402	643	784	981	1779
	F	45	395	604	718	844	1355	46	486	754	934	1052	1475	54	382	639	759	894	1165	145	382	658	779	954	1475

25% and 75% = 25th and 75th percentiles, or first and third quartiles. "LIM" = limestone forest; "SCR" = scrub forest; "RAV" = ravine forest; "LEU" = *Leucaena* stand; "SAV" = savanna complex; "URB" = urban residential.
