# Supplementary material for: Quantile regression of microgeographic variation in population characteristics of an invasive vertebrate predator
Source: PLoS One. 2017 Jun 1;12(6):e0177671. doi: 10.1371/journal.pone.0177671 (PMC5453442; doi:10.1371/journal.pone.0177671)
Supplement: S2 Table — (PDF) [file pone.0177671.s002.pdf]

S2 Table. Summary of snake mass (g) by habitat type, replicate, and sex.

		Replicate 1						Replicate 2						Replicate 3						Habitat Pooled					
Hab	Sex	N	Min	25%	Med	75%	Max	N	Min	25%	Med	75%	Max	N	Min	25%	Med	75%	Max	N	Min	25%	Med	75%	Max
LIM	M	55	14	35	49	68	297	55	9	23	42	94	907	42	7	17	32	53	159	152	7	24	42	70	907
	F	45	19	44	60	89	131	45	8	30	48	91	235	59	8	22	34	68	148	149	8	28	51	81	235
SCR	M	53	8	16	40	64	132	70	14	24	37	63	1727	57	9	20	27	54	233	180	8	21	33	62	1727
	F	51	8	24	42	73	147	30	9	24	32	75	520	43	11	20	42	77	153	124	8	22	41	74	520
RAV	M	39	6	36	57	83	1714	45	6	18	27	37	483	50	5	14	18	40	997	134	5	17	30	59	1714
	F	61	9	39	63	93	161	56	6	19	31	46	244	49	7	15	22	36	199	166	6	21	36	63	244
LEU	M	68	10	23	36	74	329	63	15	29	41	67	214	69	18	31	44	65	161	200	10	28	41	70	329
	F	33	14	25	37	55	141	36	16	27	46	73	202	31	15	27	41	72	159	100	14	26	41	71	202
SAV	M	65	7	25	43	76	279	53	7	33	66	182	774	53	5	25	53	110	1435	171	5	26	53	102	1435
	F	35	6	16	20	37	108	49	12	26	37	79	271	48	7	19	31	73	375	132	6	19	31	64	375
URB	M	55	8	23	38	62	794	54	6	29	68	254	1240	46	12	25	41	87	1307	155	6	25	41	95	1307
	F	45	7	22	33	52	428	46	15	45	92	172	667	54	7	25	37	73	233	145	7	26	43	103	667

25% and 75% = 25th and 75th percentiles, or first and third quartiles. "LIM" = limestone forest; "SCR" = scrub forest; "RAV" = ravine forest; "LEU" = *Leucaena* stand; "SAV" = savanna complex; "URB" = urban residential.
