# Supplementary material for: Quantile regression of microgeographic variation in population characteristics of an invasive vertebrate predator
Source: PLoS One. 2017 Jun 1;12(6):e0177671. doi: 10.1371/journal.pone.0177671 (PMC5453442; doi:10.1371/journal.pone.0177671)
Supplement: S3 Table — (PDF) [file pone.0177671.s003.pdf]

S3 Table. Summary of snake body condition index (CI) by habitat type, replicate, and sex.

		Replicate 1						Replicate 2						Replicate 3						Habitat Pooled					
Hab	Sex	N	Min	25%	Med	75%	Max	N	Min	25%	Med	75%	Max	N	Min	25%	Med	75%	Max	N	Min	25%	Med	75%	Max
LIM	M	55	-4.16	-1.01	-0.42	0.15	2.00	55	-1.60	-0.34	0.01	0.50	2.01	42	-2.12	-1.00	-0.46	-0.04	1.56	152	-4.16	-0.85	-0.21	0.31	2.01
	F	45	-3.65	-0.49	-0.18	0.17	1.23	45	-1.92	-0.35	0.03	0.43	1.80	59	-2.01	-0.85	-0.50	-0.12	0.98	149	-3.65	-0.68	-0.19	0.16	1.80
SCR	M	53	-1.99	-0.37	0.04	0.60	1.91	70	-1.37	-0.60	-0.21	0.41	1.67	57	-1.63	-0.57	-0.27	0.12	1.72	180	-1.99	-0.56	-0.15	0.36	1.91
	F	51	-2.24	-0.07	0.56	1.04	2.84	30	-4.62	-0.27	0.21	0.78	1.50	43	-1.43	-0.19	0.09	0.72	2.30	124	-4.62	-0.17	0.30	0.88	2.84
RAV	M	39	-2.65	-0.78	-0.26	0.32	3.35	45	-3.10	-1.36	-0.56	-0.26	0.64	50	-4.59	-1.17	-0.88	-0.45	0.83	134	-4.59	-1.17	-0.63	-0.17	3.35
	F	61	-1.57	-0.39	0.33	0.83	3.33	56	-2.20	-1.03	-0.34	-0.08	1.31	49	-4.22	-0.92	-0.62	-0.18	1.25	166	-4.22	-0.85	-0.31	0.30	3.33
LEU	M	68	-1.95	-0.65	-0.32	0.19	1.47	63	-1.63	-0.37	0.14	0.98	1.99	69	-2.65	-0.77	-0.15	0.33	1.72	200	-2.65	-0.65	-0.14	0.47	1.99
	F	33	-1.36	-0.06	0.47	0.84	2.54	36	-1.28	-0.06	0.27	0.73	1.66	31	-1.76	-0.32	0.27	0.73	1.10	100	-1.76	-0.12	0.32	0.75	2.54
SAV	M	65	-2.46	-0.48	-0.11	0.34	1.89	53	-2.70	-0.72	-0.25	0.17	2.28	53	-2.82	-0.17	0.41	1.11	2.28	171	-2.82	-0.52	-0.03	0.63	2.28
	F	35	-1.60	-0.45	-0.05	0.53	1.57	49	-1.79	-0.39	-0.11	0.53	3.49	48	-2.55	0.02	0.69	1.47	4.06	132	-2.55	-0.35	0.16	0.93	4.06
URB	M	55	-3.02	-0.82	-0.16	0.42	1.51	54	-2.33	0.27	0.78	1.75	3.72	46	-3.53	-0.76	0.06	0.69	2.33	155	-3.53	-0.55	0.25	0.81	3.72
	F	45	-2.04	-0.62	0.09	0.59	1.89	46	-1.25	0.89	1.66	2.23	3.73	54	-1.87	-0.52	0.06	0.83	3.61	145	-2.04	-0.31	0.49	1.44	3.73

25% and 75% = 25th and 75th percentiles, or first and third quartiles. Units are standard deviations from the mean of standardized residuals from a length by mass regression. “LIM” = limestone forest; “SCR” = scrub forest; “RAV” = ravine forest; “LEU” = *Leucaena* stand; “SAV” = savanna complex; “URB” = urban residential.
