# Supplementary material for: Quantile regression of microgeographic variation in population characteristics of an invasive vertebrate predator
Source: PLoS One. 2017 Jun 1;12(6):e0177671. doi: 10.1371/journal.pone.0177671 (PMC5453442; doi:10.1371/journal.pone.0177671)
Supplement: S4 Table — (PDF) [file pone.0177671.s004.pdf]

S4 Table. Summary of snake body length, mass, and condition index pooled by forest and non-forest habitat types and by sex.

			Snout-vent Length (mm)							Mass (g)							Condition Index (sd)						
Hab	Sex	N	Min	5%	25%	Med	75%	95%	Max	Min	5%	25%	Med	75%	95%	Max	Min	5%	25%	Med	75%	95%	Max
Forest	M	666	354	466	618	753	907	1169	1950	5	11	23	37	65	164	1727	-4.59	-1.63	-0.77	-0.27	0.28	1.26	3.35
	F	539	378	485	633	768	917	1057	1404	6	11	24	41	73	130	520	-4.62	-1.30	-0.53	-0.03	0.58	1.30	3.33
Non-forest	M	326	350	487	650	805	990	1479	1876	5	12	25	46	101	532	1435	-3.53	-1.44	-0.53	0.04	0.74	2.19	3.72
	F	277	370	482	603	743	913	1142	1475	6	13	22	37	79	247	667	-2.55	-1.24	-0.34	0.27	1.14	2.46	4.06
