# Supplementary material for: Quantile regression of microgeographic variation in population characteristics of an invasive vertebrate predator
Source: PLoS One. 2017 Jun 1;12(6):e0177671. doi: 10.1371/journal.pone.0177671 (PMC5453442; doi:10.1371/journal.pone.0177671)
Supplement: S4 Dataset — (DOCX) [file pone.0177671.s008.docx]

Metadata (column name explanations) for data provided with manuscript “Quantile regression of microgeographic variation in population characteristics of an invasive vertebrate predator” by Siers, Savidge, & Reed

S1 Dataset.csv = Original snake capture data plus field added for analysis and plotting purposes

| [blank] | Row names |
| --- | --- |
| PITTAG | Unique specimen identifier |
| IWVISLOCALE | Original site code: ACAR=LEU1, AGH=URB3, ASOU=SCR1, DEDU=URB2, FB3R=SAV2, HMUR=LIM2, IHAS=SAV3, MALR=LEU3, MTRS=SAV1, NMGB=LIM3, NSOP=LEU2, NWFO=LIM1, OARF=RAV2, OTGC=RAV3, PAGR=RAV1, TALO=SCR3, TZFS=Included with FB3R in SAV2, YIGO=URB1, YONA=SCR2 |
| Date | Date snake was captured |
| OBSERVER | Initials of biologist capturing snake |
| MORPHED.BY | Initials of observer collecting morphometrics in the field |
| SVL | Snout-vent length as measured in the field (mm) |
| WEIGHT | Snake mass as measured in the field (g) |
| SEX | Sex as probed in the field |
| SEAS | Season (wet/dry) of snake capture |
| NO | Site number for ordering purposes |
| SIT | Three-character site code: AGH=URB3, COM=LEU1, DED=URB2, HMU=LIM2, IHA=SAV3, MAL=LEU3, MAR=SCR1, MTR=SAV1, NWF=LIM1, OAL=LIM3, ORA=RAV2, OAS=SAV2, OPT=LEU2, OTG=RAV3, PAG=RAV1, TAL=SCR3, YIG=URB1, YON=SCR2 |
| HAB | Habitat type code |
| HABITAT | Habitat type spelled out |
| PLOTORD | Sequence for ordering sites in plots |
| NAME | Site name spelled out |
| COL | Color for habitats used in plots |
| PCH | Symbol for habitats used in plots |
| NECSVL | Snout-vent length measured on necropsy (mm) |
| NECWT | Weight measured in necropsy (g) |
| NECBY | Initials of biologist performing necropsy |
| FOR | Forest versus savanna and urban classification |
| FOR2 | Same as above but considering ravine forest as separate forest type |
| PREYWT | Mass (g) of prey in stomach contents |
| ADJWT | Adjusted snake mass (g) after subtracting PREYWT |
| CI | Body condition index |
| STOM | Indicator (Y/N) of presence of stomach contents |

S2 Dataset.csv = This summarized subset of data was used for the quantile regression models

| [blank] | Row names based on specimen unique identifier |
| --- | --- |
| svl | Snout-vent length (mm) |
| hab | Habitat code |
| sex | Sex as confirmed by necropsy |
| seas | Season (wet/dry) specimen was collected |
| site | Three-character site code: AGH=URB3, COM=LEU1, DED=URB2, HMU=LIM2, IHA=SAV3, MAL=LEU3, MAR=SCR1, MTR=SAV1, NWF=LIM1, OAL=LIM3, ORA=RAV2, OAS=SAV2, OPT=LEU2, OTG=RAV3, PAG=RAV1, TAL=SCR3, YIG=URB1, YON=SCR2 |
| ci | Body condition index |
| stom | Binary indicator (Y/N) of stomach contents presence |
| for1 | Habitat consolidation lumping all forest types into one “FOR” class |
| for2 | Alternate consolidation considering ravine forest as separate forest type |
| wt | Snake mass (g) |
| ahab | Alternate habitat classification coding URB as AAA to set a different level as the one to be calculated by subtraction (allows plotting of all classes simultaneously) |
| asit | Alternative site classification (as above) |
| aseas | Alternative season classification (as above) |
| sexsit | Classification of site by sex |
| sexfor | Classification of sex by forest |
| asexhab | Alternative classification of sex by habitat |
| asexsit | Alternative classification of sex by site |
| rep | Replicate number as referenced in manuscript |

S3 Dataset.csv = Prey observation data collected during visual surveys

| IWVISLOCALE | Original site code: ACAR=LEU1, AGH=URB3, ASOU=SCR1, DEDU=URB2, FB3R=SAV2, HMUR=LIM2, IHAS=SAV3, MALR=LEU3, MTRS=SAV1, NMGB=LIM3, NSOP=LEU2, NWFO=LIM1, OARF=RAV2, OTGC=RAV3, PAGR=RAV1, TALO=SCR3, TZFS=Included with FB3R in SAV2, YIGO=URB1, YONA=SCR2 |
| --- | --- |
| Date | Date observation was made |
| OBSERVER | Initials of biologist making prey observation |
| SPECIES_ADDED | Species code for prey observations: AC=*Anolis carolinensis*; AE=avian egg; AV=avian (unidentified bird); BAT=*Pteropus mariannus*; BBQU=*Colinus virginianus*; BLFR=*Francolinus francolinus*; CAT=*Felis catus*; CF=*Carlia ailanpala*i; DOFO=*Gallus gallus domesticus*; EC=*Emoia cearuleocaud*a; EP=*Eleutherodactylus planirostris*; ETSP=*Passer montanus*; FC=*Fejervarya cancrivora*; FR=unidentified frog; GE=unidentified gecko; GM=*Gehyra mutilata*; HF=*Hemidactylus frenatus*; HG=*Hylarana guentheri*; LF=*Litoria fallax*; LL=*Lepidodactylus lugubris*; MM=*Mus musculus*; MP=*Microhyla pulchra*; NP=*Nactus pelagicus*; PHTD=*Streptopelia bitorquata*; PM=*Polypedates megacephalus*; RB=*Ramphotyphlops braminus*; RD=unidentified rodent; RT=unidentified rat; SK=unidentified skink; SM=*Suncus murinus*; VI=*varanus indicus* |
| TIME | Time of observation |
